# The Interplay Between Redox Status, Exercise Intensity, and Mucosal Immunity: Toward Context-Dependent Antioxidant Strategies

**DOI:** 10.3390/antiox15101241

**Published:** 2026-09-25

**Authors:** Yousra Alsinani, Hossein Shirvani, Majid AL-Busafi, Amr Mahmoud Abu El-Fadl Ibrahim

**Affiliations:** 1 Curriculum and Instruction Department, College of Education, Sultan Qaboos University, Muscat 123, Oman; 2 Exercise Physiology Research Center, Life Style Institute, Baqiyatallah University of Medical Sciences, Tehran 14359-16471, Iran; 3 Physical Education Department, College of Education, Sultan Qaboos University, Muscat 123, Oman; 4 Gymnastics and Exercise Training Department, Faculty of Sports Sciences, Matrouh University, Mersa Matruh 51511, Egypt

**Keywords:** exercise intensity, redox biology, oxidative stress, salivary IgA, mucosal immunity, antioxidants, sports nutrition, personalized nutrition, recovery

## Abstract

Exercise simultaneously challenges redox homeostasis, neuroendocrine regulation, metabolism, and immune function. The response is strongly dependent on exercise dose, recovery, nutritional status, and individual characteristics. This perspective updates the evidence on the relationship among exercise intensity, redox biology, and mucosal immunity, with particular emphasis on salivary secretory immunoglobulin A (sIgA) and antioxidant strategies. Current evidence supports a hormetic interpretation: exercise-derived reactive oxygen and nitrogen species (ROS/RNS) are not intrinsically harmful and participate in adaptive signaling, whereas excessive or poorly recovered exposure can contribute to oxidative damage, inflammation, impaired recovery, and transient changes in immune function. A 2024 randomized crossover study in 12 untrained young men found that the 75% VO_2_max condition produced a significantly lower percentage change in sIgA secretion rate compared with the 55% VO_2_max condition, while lower-intensity or shorter-duration trials did not. Importantly, this finding should be interpreted as a protocol-specific observation rather than evidence of a universal biological threshold. Salivary oxidative-stress evidence is also heterogeneous, and the quality of evidence remains low. Contemporary antioxidant guidance increasingly favors food-first approaches and selective supplementation for nutrient inadequacy or periods of unusually high training stress rather than routine high-dose antioxidant use. Evidence for specific interventions is mixed: astaxanthin, β-glucans, sesame, and polyphenol formulations show promising but still limited human evidence, while a 2024 trial in adults with obesity illustrates that melatonin, a pleiotropic redox-modulating molecule, can produce context-dependent effects. The most defensible personalized framework therefore combines training load, recovery, dietary adequacy, mucosal-immune monitoring, and—where clinically justified—redox phenotyping. Genetic and multi-omics approaches remain research tools rather than established clinical decision rules.

## 1. Introduction

Regular exercise produces broad health benefits, but the biological response depends on dose and context. Exercise increases flux through mitochondrial and non-mitochondrial redox pathways, alters endocrine signaling, and transiently redistributes immune cells. The traditional view that exercise-generated ROS simply cause oxidative damage has been replaced by a more nuanced redox-signaling model. Moderate reactive-species signaling can activate endogenous antioxidant and stress-response pathways, whereas excessive exposure or inadequate recovery may increase the oxidative modification of lipids, proteins, and nucleic acids and contribute to impaired recovery [1,2,3,4].

Mucosal immunity provides a particularly useful window into exercise-related physiological stress because saliva can be sampled repeatedly with minimal burden. Secretory IgA is an important component of upper-airway defense, but its interpretation requires care: sIgA varies with saliva flow, hydration, circadian timing, nutritional status, psychological stress, environmental conditions, and sampling methodology. Early work by Gleeson and colleagues established the association between strenuous training, transient reductions in salivary immunoglobulins, and respiratory-illness risk, while also emphasizing substantial methodological and biological variability [5,6,7].

The central question is therefore not whether exercise is good or bad for immunity or whether ROS should be eliminated. Rather, the relevant question is how exercise dose, recovery, nutritional status, and individual redox phenotype interact to determine whether reactive-species signaling remains adaptive or becomes excessive. This distinction is essential when considering antioxidant supplementation.

The principal conceptual contribution of this perspective is not the proposal that antioxidant strategies should be individualized—an idea that has been discussed previously—but rather the integration of four interacting dimensions within a single framework: exercise dose, redox response, mucosal immunity, and recovery context. This integrative model is intended to organize future research and monitoring and to clarify where current evidence supports intervention and where it does not.

This revised perspective has three objectives: (1) integrate current human evidence on exercise dose, redox responses, and mucosal immunity; (2) critically reassess antioxidant interventions without assuming that every published intervention is beneficial; and (3) develop a more conservative, evidence-based framework for personalized antioxidant use.

### Literature Search Strategy

This perspective was developed through a narrative synthesis of human evidence published between 2000 and 2026. Sources were identified through targeted searches of PubMed, Scopus, and Web of Science using combinations of the following terms: *exercise intensity*, *redox*, *oxidative stress*, *salivary IgA*, *mucosal immunity*, *antioxidants*, *astaxanthin*, *β-glucans*, *polyphenols*, *sesame*, *melatonin*, and *personalized nutrition*. Priority was given to human randomized controlled trials, crossover studies, systematic reviews, and position stands. Narrative reviews were used for contextual background. Animal studies were excluded. The nutritional interventions discussed were selected because they represent distinct mechanistic categories (direct antioxidants, indirect redox modulators, immunomodulatory nutraceuticals, and pleiotropic signaling molecules) and because they have been evaluated in human exercise studies. The evidence for each intervention is described qualitatively as preliminary, limited, or moderate where appropriate.

## 2. Exercise Intensity, Redox Signaling, and the Eustress–Distress Continuum

### 2.1. Hormesis as the Conceptual Framework

The concept of hormesis provides the central conceptual framework for this perspective. Hormesis describes a biphasic dose–response relationship in which a low-dose stimulus produces beneficial adaptive effects, whereas a high-dose stimulus produces detrimental effects. In the context of exercise, moderate reactive oxygen and nitrogen species (ROS/RNS) production acts as a hormetic stimulus that activates endogenous antioxidant and stress-response pathways, leading to improved redox resilience and metabolic adaptation. In contrast, excessive or poorly recovered exposure can overwhelm these adaptive mechanisms, resulting in oxidative distress and mucosal immune perturbation. This hormetic framework integrates the apparently contradictory observations that exercise-derived ROS are both necessary for adaptation and potentially harmful when produced in excess. It also provides a biologically coherent rationale for why indiscriminate antioxidant supplementation may be counterproductive: by blunting the hormetic stimulus, such interventions may attenuate the very adaptations that exercise is intended to produce.

This hormetic continuum is not defined by a single VO_2_max percentage or exercise duration. Rather, it reflects the dynamic interplay between exercise dose, individual redox phenotype, mucosal immune status, and recovery context. The transition from adaptive eustress to maladaptive distress is individual and context-dependent, and it cannot be reduced to a universal threshold.

### 2.2. Redox Signaling Rather than Simple Oxidative Damage

Exercise increases ROS/RNS generation through several sources, including mitochondrial electron transport, NADPH oxidases, xanthine oxidase, and inflammatory-cell pathways. These species can modify signaling proteins and transcriptional pathways involved in mitochondrial biogenesis, antioxidant defense, metabolism, and adaptation. Consequently, the suppression of all exercise-related ROS is neither biologically desirable nor supported by current evidence [2,3,4,8].

The practical distinction is between transient redox signaling and sustained oxidative distress. The latter is better conceptualized as an imbalance in which oxidant production, antioxidant buffering, repair, and recovery become mismatched. Importantly, commonly used biomarkers such as total antioxidant capacity, FRAP, d-ROMs, malondialdehyde, protein carbonyls, or individual enzyme activities do not measure one single construct called oxidative stress. They represent different aspects of redox biology and should not be interpreted interchangeably [3,9].

### 2.3. Exercise Dose Matters, but There Is No Universal Threshold

The strongest recent evidence for an exercise-dose effect on mucosal immunity comes from a randomized crossover study [10]. In this study, 12 healthy untrained young men completed 30 min of cycling at 35%, 55%, and 75% VO_2_max, and separately, 30, 60, and 90 min at 55% VO_2_max. The 75% VO_2_max condition produced a significantly lower percentage change in sIgA secretion rate compared with the 55% VO_2_max condition (45.7% lower), while the 90-min condition at 55% VO_2_max produced a 37.0% lower percentage change than the 30-min condition. Salivary cortisol increased markedly after the 75% VO_2_max trial. It should be emphasized that these values represent differences in the percentage change between exercise conditions, not absolute reductions from the baseline. The absolute magnitude of sIgA change from pre- to post-exercise requires separate consideration. Beyond mucosal outcomes, high-intensity interval training (HIIT) has also been shown to confer cardiovascular protection through the upregulation of reverse cholesterol transport elements, including increased hepatic and intestinal ABCA1 expression and elevated plasma HDL levels, suggesting that HIIT’s biological effects extend across multiple physiological systems relevant to long-term health [11].

These results are useful because intensity and duration were manipulated within the same participants. However, the findings should not be converted into a universal clinical threshold. The sample was small, exclusively male, young, untrained, and studied during acute cycling. The investigators themselves note that hydration, circadian rhythm, nutrition, saliva-collection methods, and exercise modality can influence sIgA responses [10]. Thus, ≥75% VO_2_max for 30 min or ≥90 min at 55% VO_2_max should be described as experimental conditions associated with transient mucosal perturbation in this specific population and protocol—not as a universal red line requiring antioxidant treatment.

This qualification is consistent with the broader mucosal-immunity literature, which has long shown that responses differ according to training status, exercise characteristics, environment, nutritional state, and individual susceptibility [5,6,7].

## 3. Mucosal Immunity and Salivary Biomarkers

### 3.1. sIgA as a Stress-Sensitive Marker

sIgA is a major antibody at mucosal surfaces and has been widely studied in exercise immunology. Earlier reviews reported transient post-exercise reductions following strenuous exercise and associations between chronically low salivary immunoglobulin levels and respiratory illness in some athletic populations [5,6]. These observations justify sIgA as a candidate monitoring biomarker, but they do not establish that every acute reduction causes infection or that raising sIgA necessarily prevents infection.

The 2024 dose–response study adds important experimental detail but also reinforces the need for standardized measurement. The outcome was secretion rate rather than concentration alone, and the response differed with both exercise intensity and duration [10]. For longitudinal athlete monitoring, flow rate, sampling time, hydration, recent food/fluid intake, and illness status should therefore be documented rather than treating a single sIgA value as an independent diagnostic marker.

A decrease in salivary sIgA is a mucosal immune biomarker change. It does not automatically demonstrate generalized immunosuppression, nor does it by itself establish increased infection incidence. These distinctions should be maintained when interpreting sIgA data and when considering nutritional or monitoring interventions.

Salivary sIgA can be expressed as concentration (μg/mL), secretion rate (μg/min), or as a ratio to total protein. These variables are physiologically related but not interchangeable. Concentration is influenced by salivary flow rate, whereas secretion rate incorporates flow and is generally considered a more robust measure of mucosal output. Salivary flow rate itself varies with hydration, autonomic activity, circadian timing, and collection method. Studies should therefore report how sIgA was measured and normalized, and comparisons across studies should account for these methodological differences.

### 3.2. Saliva as a Redox and Immune Matrix

Saliva is attractive because it can provide both immune and redox information. A 2022 systematic review identified 22 eligible exercise studies and found that 14 reported increases in salivary antioxidant parameters while eight reported increased lipid peroxidation. However, heterogeneity was substantial and the GRADE assessment indicated very low certainty of evidence [9]. The authors concluded that exercise-related increases in both oxidative-stress and antioxidant activity in saliva may occur, but methodological differences make broad conclusions difficult.

A major methodological challenge in salivary sIgA research is the confounding effect of salivary flow rate. sIgA concentration is inversely related to saliva flow: as flow increases, concentration tends to decrease, even when absolute secretion is unchanged. This relationship can produce spurious associations between exercise and sIgA when concentration alone is reported. Expressing sIgA as a secretion rate (concentration × flow rate) partially addresses this issue and is generally preferred. However, flow rate itself varies with hydration, autonomic activity, circadian timing, collection method, and exercise intensity, and it may not be fully normalized by simple multiplication. An alternative approach is to express sIgA relative to a flow-dependent marker such as bicarbonate or total protein, which may further reduce flow-related variability. Bicarbonate is a flow-dependent component of saliva that correlates closely with acinar secretion and has been proposed as a normalization factor. To date, however, no single normalization method has been universally adopted, and direct comparisons across studies remain difficult. Future studies should report both concentration and secretion rate, document flow rate and collection methodology, and consider flow-dependent normalization where feasible.

This is directly relevant to personalized strategies: salivary redox measures should be treated as complementary signals rather than definitive estimates of whole-body oxidative stress. A more rigorous monitoring panel would combine standardized sIgA secretion rate with selected redox markers, training load, recovery indices, and clinical symptoms.

### 3.3. Environmental and Contextual Modifiers

Environmental conditions can modify mucosal responses independently of exercise dose. Mylona et al. reported different sIgA responses to the same moderate exercise protocol in cold versus thermoneutral conditions [12]. Earlier work in endurance athletes and combat-sport athletes also suggests that cumulative training load and rapid weight loss can influence mucosal and immune measures [13,14]. Psychological stress, sleep quality, recovery status, and recent illness may also modify exercise-related mucosal immune responses. These factors should be considered alongside physical training metrics in monitoring protocols. However, the mechanistic pathways linking neuroimmune modulation to salivary IgA remain to be established, and findings from post-infectious populations should not be generalized to healthy athletes without qualification [15,16].

The “open-window” concept proposes that selected immune parameters are transiently modified after strenuous exercise. However, the commonly cited 3–72 h window encompasses highly heterogeneous immune phenomena, and transient changes in immune variables do not necessarily represent clinically meaningful immunosuppression or increased infection risk. These findings argue against attributing every change in sIgA to exercise intensity alone.

## 4. What Human Evidence Actually Shows About Nutritional and Redox-Modulating Interventions

The interventions discussed in this section are not mechanistically equivalent. They can be broadly classified as follows:

- **Direct antioxidants**: Compounds that directly scavenge ROS/RNS (e.g., astaxanthin).
- **Indirect redox modulators**: Compounds that modulate endogenous antioxidant signaling pathways (e.g., polyphenols).
- **Immunomodulatory nutraceuticals**: Compounds whose primary action is immune modulation rather than direct redox chemistry (e.g., β-glucans).
- **Pleiotropic signaling molecules**: Compounds with context-dependent redox, endocrine, and immune effects (e.g., melatonin).
- **Whole-food interventions**: Dietary patterns or foods containing multiple bioactive compounds (e.g., sesame).

This classification is used throughout the section to improve mechanistic precision and to avoid treating these interventions as interchangeable.

### 4.1. The Blunting Problem

The modern antioxidant literature does not support the assumption that more antioxidant capacity is always better. A 2020 review of antioxidant supplements and endurance exercise concluded that many compounds have insufficient evidence for routine use and highlighted the possibility that chronic antioxidant exposure can interfere with exercise-induced signaling [8]. In particular, the review noted that chronic combined vitamin C and E supplementation during heavy endurance training is not recommended because of potential interference with skeletal-muscle adaptation.

An early mechanistic trial illustrates why this issue matters. Vassilakopoulos et al. administered a combination of vitamins E, A, and C, allopurinol, and N-acetylcysteine to six untrained adults and found that antioxidant treatment markedly blunted exercise-induced TNF-α, IL-1β, and IL-6 responses [17]. This study was small and used a pharmacologically intensive combination, so it should not be generalized to ordinary dietary antioxidant intake. Nevertheless, it demonstrates that suppressing redox-sensitive signaling is biologically consequential.

It is important not to equate the attenuation of an acute exercise-induced IL-6 response with impaired physiological adaptation. Exercise-induced IL-6 functions as a myokine with metabolic roles that differ substantially from chronically elevated inflammatory IL-6. The functional consequences of blunting acute cytokine responses require the direct measurement of training adaptation outcomes rather than inference from cytokine concentrations alone.

Conversely, Robson et al. found that seven days of vitamin C, vitamin E, and β-carotene supplementation increased post-exercise neutrophil oxidative burst in 12 endurance-trained participants [18]. The finding illustrates why antioxidant effects should be judged by the specific physiological endpoint rather than assumed from a label or from changes in a single oxidative-stress marker.

### 4.2. Astaxanthin: Promising but Not Definitive

Baralić et al. (2015) randomized 40 trained male soccer players to 4 mg/day astaxanthin or placebo for 90 days and reported higher resting sIgA and lower prooxidant–antioxidant balance in the astaxanthin group, together with the attenuation of some inflammatory and muscle-damage measures [19]. This is relevant evidence, but it is a single relatively small study and should not be described as definitive proof that astaxanthin prevents mucosal immune suppression.

A separate 2025 study in trained water-polo players examined short-term astaxanthin supplementation together with cold-water immersion after high-intensity interval exercise. The article is indexed in EBSCO with a DOI and reports serum IgA and IL-6 outcomes [20]. Because this study combines supplementation with recovery treatment and is not directly equivalent to a mucosal sIgA trial, its findings should be treated as supportive rather than as confirmation of a specific antioxidant effect on salivary immunity.

### 4.3. β-Glucans and Micronutrients

An eight-week randomized, double-blind, placebo-controlled study [21] in 39 healthy moderately active men used a yeast-cell-derived formulation containing β-glucans and micronutrients. The supplemented group showed higher fasting FRAP and higher superoxide dismutase (SOD) activity at 60 min after HIIT, alongside reductions in TNF-α and reported improvements in upper-respiratory outcomes. This trial provides useful evidence connecting redox and immune endpoints in healthy moderately active men. However, the formulation contained multiple components, so the effects cannot be attributed to β-glucans alone.

### 4.4. Polyphenols and the Emerging Multi-Omics Literature

A 2026 randomized study by Huang et al. investigated a high-polyphenol formulation containing quercetin, EGCG, resveratrol, and curcumin in 80 adolescent athletes during 12 weeks of high-intensity training. The intervention was associated with preservation of CD4^+^/CD8^+^ ratios, favorable Treg/Th17 changes, multi-omics signatures involving the NF-κB, AMPK-mTOR and Nrf2 pathways, and maintenance of mucosal immunity [22]. This is a notable new study because it integrates immune, metabolic, and microbiome measurements. Nevertheless, the population was adolescent athletes and the intervention was a multi-compound formulation; extrapolation to adult athletes or to individual polyphenols is premature.

### 4.5. Sesame

A 2026 systematic review by Jafari et al. identified four randomized controlled trials involving 140 semiprofessional athletes. Across 4–10 week interventions, sesame supplementation was associated with higher GPx and SOD, lower MDA, and changes in inflammatory markers. The authors also noted that some cytokine changes appeared primarily attributable to exercise rather than sesame [23]. This distinction is important: the evidence supports sesame as a promising nutritional adjunct, but it does not establish a mucosal-immunity-specific benefit.

### 4.6. Melatonin as a Context-Dependent Example

A 2024 randomized controlled trial [24] provides an important cautionary example. In 18 adults with obesity undergoing a two-week multidisciplinary weight-reduction program, 2 mg/day melatonin was associated with higher post-exercise d-ROMs and erythrocytic glutathionyl hemoglobin and prevented the reduction in IL-6 observed in the placebo group. The trial investigators interpreted melatonin as having conditional pro-oxidant effects in this context. This small study should not be used to claim that melatonin is generally harmful, but it demonstrates why antioxidant recommendations cannot be detached from metabolic context.

### 4.7. Summary Table of Key Human Studies

Table 1 summarizes the key human studies discussed in this review.

## 5. Updated Conceptual Model

The evidence supports a continuum rather than a rigid three-zone model. At one end, low-to-moderate exercise generally permits redox homeostasis and adaptive signaling. With increasing intensity, duration, environmental stress, energy deficit, or insufficient recovery, the probability of transient oxidative and immune perturbation increases. At the extreme end, repeated high-load exposure can exceed recovery capacity. The transition is individual and dynamic; it is not defined by a single VO_2_max percentage.

A useful conceptual model therefore has four interacting dimensions: (1) exercise dose, including intensity, duration, frequency, and accumulated load; (2) redox phenotype, including dietary adequacy and selected biomarkers; (3) mucosal phenotype, particularly longitudinal sIgA secretion patterns; and (4) recovery context, including sleep, hydration, energy availability, environmental exposure, travel, illness, and competition density. Antioxidants are one possible modifier within this system, not the central treatment for all exercise-induced stress.

This four-dimensional framework is illustrated in Figure 1.

Figure 1 shows the conceptual model illustrating the integrative framework proposed in this perspective. External exercise dose (intensity, duration, frequency, accumulated load) interacts with individual internal response domains (redox response, neuroendocrine response, mucosal immune response) and is modified by training status, sleep and recovery, psychological stress, metabolic health, and nutritional status. The balance between adaptive eustress and persistent maladaptive distress determines the physiological outcome. Management implications include a food-first approach and selective supplementation only when appropriately justified. The hormetic continuum is depicted, with the transition from adaptive eustress to maladaptive distress being individual and context-dependent.

Figure 1 synthesizes the evidence reviewed in this perspective into a single conceptual model. The model emphasizes that exercise dose is the external input, but the physiological outcome is determined by the interaction between individual redox, neuroendocrine, and mucosal immune responses, as modified by training status, recovery, psychological stress, metabolic health, and nutritional status. The hormetic balance point determines whether the exercise stimulus produces adaptive eustress or maladaptive distress. This framework is hypothesis-generating and intended to organize future research and monitoring, not to represent a validated diagnostic or prescribing algorithm.

## 6. Toward Context-Dependent Antioxidant Strategies

### 6.1. Operational Definitions: What Personalization Can and Cannot Mean

In this perspective, we use the following operational definitions:

- **Redox phenotype**: A longitudinal intra-individual profile comprising baseline redox markers (e.g., FRAP/TAC, SOD, GPx, and where available lipid-peroxidation markers), the acute redox response to a standardized exercise stimulus, and recovery kinetics over 24–72 h. A single measurement is not considered sufficient to define a phenotype.
- **Mucosal phenotype**: A longitudinal intra-individual profile comprising baseline salivary sIgA concentration and secretion rate, salivary flow rate, the acute sIgA response to exercise, and recovery kinetics, interpreted alongside recent illness history and training load. A single sIgA value is not considered a phenotype.
- **Personalization**: The use of these longitudinal profiles, together with training load, dietary adequacy, and recovery context, to inform individualized monitoring and—where evidence supports it—selective nutritional intervention. Personalization in this manuscript does not refer to genotype-guided or biomarker-guided antioxidant prescribing, which remains investigational.

These definitions are intended to organize future research and monitoring, not to constitute a validated clinical protocol. Analytical and biological variability in redox and mucosal biomarkers remains substantial, and the threshold values, sampling intervals, and decision rules required for clinical or performance application have not been established.

### 6.2. What Personalization Should Mean in Practice

Personalization should begin with assessment rather than supplementation. The concept of redox phenotyping was proposed explicitly in the personalized-nutrition literature as a way to identify people who may be more likely to benefit from antioxidant intervention and to avoid unnecessary treatment [25]. The 2026 ISSN position stand similarly emphasizes that antioxidant effects depend on dose, timing, compound, training status, diet, baseline antioxidant capacity, and the athlete’s specific recovery needs [26].

In practical terms, the first intervention should be adequate energy intake, sufficient protein and carbohydrate availability, a varied diet rich in fruits, vegetables, whole grains, legumes, nuts, seeds, and other polyphenol-containing foods, appropriate hydration, and adequate recovery. Supplementation becomes more defensible when a dietary inadequacy or deficiency is documented, when food intake cannot meet the requirements, or during unusually high periods of training distress [26].

### 6.3. A Conservative Monitoring Framework

These steps are proposed as a conceptual monitoring framework. They have not been prospectively validated and should not be interpreted as a standardized clinical or sports-nutrition protocol.

Step 1—Characterize training exposure: intensity distribution, duration, frequency, recent load changes, competition density, and recovery days.

Step 2—Screen for modifiable stressors: energy availability, carbohydrate intake, hydration, sleep, travel, environmental extremes, rapid weight loss, and intercurrent illness.

Step 3—Establish longitudinal mucosal data: use standardized sIgA secretion-rate sampling rather than relying on a single concentration measurement.

Step 4—Use redox biomarkers selectively: interpret FRAP/TAC, SOD, GPx, lipid-peroxidation markers, or other assays as complementary measures, not interchangeable definitions of “oxidative stress”.

Step 5—Correct deficiencies before adding pharmacological doses: food-first strategies and clinically indicated micronutrient correction should precede routine high-dose antioxidant cocktails.

Step 6—Match intervention to objective: training adaptation, recovery, competition support, or correction of nutritional inadequacy are different goals and should not receive identical antioxidant strategies.

Step 7—Reassess response: changes in symptoms, recovery, performance, sIgA trajectory, and selected biomarkers should determine whether an intervention is continued.

### 6.4. Genetic and Multi-Omics Personalization: Promising, Not Yet Ready for Routine Use

Genetic polymorphisms in SOD2, GPX1, CAT, and NFE2L2 have been proposed as potential modifiers of redox biology, but there is not sufficient clinical evidence to use these polymorphisms as routine decision rules for antioxidant supplementation. Multi-omics studies, including the 2026 adolescent-athlete polyphenol study, are better viewed as hypothesis-generating tools at present [22].

### 6.5. Timing and Training Adaptation

The strongest general principle is to avoid chronic, indiscriminate high-dose antioxidant use during adaptive training. This does not mean that antioxidants should never be used. Rather, the intervention should be matched to the athlete’s nutritional status and objective. Rather than being absolutely contraindicated, chronic high-dose antioxidant supplementation may attenuate selected exercise-induced adaptations under certain conditions, depending on compound, dose, timing, nutritional status, training status, and the outcome measured. A redox-informed framework for training adaptation similarly emphasizes that antioxidant strategies should be aligned with the specific physiological phase of training and recovery rather than applied uniformly across the training cycle [27]. The 2026 ISSN position stand favors whole foods and reserves supplementation primarily for insufficiency, inadequate dietary intake, or periods of unusually high stress [26].

## 7. Discussion

The present synthesis of human evidence from 2000 to 2026 reveals that the relationship between exercise, redox biology, and mucosal immunity is fundamentally governed by dose–response dynamics, recovery capacity, and individual metabolic context. While the preceding sections have summarized the experimental findings, a deeper mechanistic integration is necessary to translate these observations into actionable physiological principles. The discussion that follows examines five interconnected mechanistic pillars: neuroendocrine–redox crosstalk, mitochondrial adaptation signaling, gut–mucosal barrier integrity, compound-specific antioxidant pathways, and the emerging role of neuroimmune modulation.

Throughout this discussion, we distinguish among four levels of evidence:

1. Directly observed findings in the cited human studies;
2. Mechanisms supported independently by experimental literature;
3. Plausible mechanistic interpretations (biologically consistent but not directly tested in the exercise context);
4. Hypothesis-generating proposals for future research.

### 7.1. Neuroendocrine–Redox Crosstalk and the Cortisol–IgA Axis

The most robust experimental evidence for intensity-dependent mucosal perturbation comes from Uchino et al., who directly observed that the 75% VO_2_max condition produced a significantly lower percentage change in sIgA secretion rate compared with the 55% VO_2_max condition, concomitant with a 107.6% increase in salivary cortisol [10]. Mechanistically, this association is consistent with—but not proof of—the following proposed pathway. Cortisol exerts immunosuppressive effects through glucocorticoid receptor (GR) binding on B-lymphocytes and plasma cells resident in mucosal-associated lymphoid tissue (MALT). The *PIGR* gene, which encodes the polymeric immunoglobulin receptor (pIgR) responsible for the transcytosis of dimeric IgA across epithelial cells, contains a functional glucocorticoid response element, and glucocorticoids have been shown to transcriptionally regulate pIgR expression [28]. However, the direction of this regulation is tissue- and context-dependent: glucocorticoids upregulate pIgR mRNA in the intestinal epithelium [28], whereas corticosteroid exposure has been associated with altered pIgR/Fc receptor expression and reduced immunoglobulin transport in other mucosal contexts [29,30]. Whether GR activation suppresses or otherwise modifies pIgR expression in MALT during exercise has not been directly examined, and the specific direction proposed here should therefore be regarded as hypothesis-generating. Concurrently, high-intensity exercise increases sympathetic neural outflow, releasing norepinephrine, which binds β2-adrenergic receptors on salivary acinar cells. β-Adrenergic signaling is a well-established regulator of salivary acinar secretory activity, and β-adrenergic agonists activate CREB and stimulate protein secretion in parotid acinar cells [31]. This may reduce the saliva flow rate independently of cortisol—a dual-hit mechanism that could lower sIgA concentration not solely through reduced synthesis but also through diminished fluid-phase transport. The negative correlation observed between salivary cortisol and saliva flow rate in the Uchino study (r = −0.52, *p* < 0.01) is consistent with this neuroendocrine–exocrine interplay [10]. Because neither GR activation nor PIGR expression was measured in the Uchino et al. study, this pathway should be regarded as a hypothesis-generating integration of independent mechanistic evidence rather than as a demonstrated explanation of the observed sIgA response.

An important limitation of this proposed mechanism is its temporal scale. The observed reductions in salivary sIgA occurred immediately after exercise, whereas transcriptional suppression of *PIGR* and downstream changes in IgA transcytosis would be expected to operate over hours to days. Immediate post-exercise changes in salivary IgA are therefore more likely to reflect acute autonomic regulation of salivary flow, secretion kinetics, and the redistribution of existing immunoglobulin pools. Transcriptional mechanisms involving reduced PIGR expression may contribute to the persistence or magnitude of suppression over longer recovery windows, but they are unlikely to explain the immediate response. This distinction should be considered when interpreting acute versus longitudinal sIgA data.

It should be noted that proposing glucocorticoid receptor activation as a mechanism of IgA suppression and subsequently invoking oxidative modification of the receptor as a cause of functional glucocorticoid resistance may appear contradictory. These two mechanisms are not mutually exclusive, but they operate in different physiological contexts and on different time scales. Acute GR activation by exercise-induced cortisol may transiently suppress PIGR transcription, whereas oxidative modification of GR cysteine residues during sustained oxidative distress may attenuate GR sensitivity during recovery or overreaching. The temporal sequence and relative contribution of these mechanisms remain to be determined and should be considered hypothesis-generating.

### 7.2. Mitochondrial Signaling, Nrf2 Redox Homeostasis, and the Blunting Paradox

The hormetic model presented in Section 2.1 requires mechanistic grounding in mitochondrial biology. Exercise-induced ROS are not merely metabolic byproducts; they are essential second messengers for the activation of AMPK and the transcriptional co-activator PGC-1α, which orchestrate mitochondrial biogenesis and oxidative substrate flexibility [4,8]. A key downstream effector is Nrf2, which dissociates from its cytosolic inhibitor Keap1 upon ROS-mediated oxidation of specific cysteine thiols. It has been proposed, based on independent cell-biology literature, that Nrf2 then translocates to the nucleus, driving the transcription of over 200 cytoprotective genes, including SOD, GPx, and heme oxygenase-1 (HO-1). This endogenous amplification system is the mechanistic cornerstone of “oxidative eustress” [3,22].

However, the high-polyphenol intervention by Huang et al. (2026) illustrates that exogenous redox modifiers can either support or blunt this pathway [22]. In their adolescent-athlete cohort, polyphenols appeared to synergize with Nrf2 signaling, preserving CD4^+^/CD8^+^ ratios [22]. In contrast, the early trial by Vassilakopoulos et al. (2003) used high-dose vitamins E, A, C, allopurinol, and N-acetylcysteine [17]. One possible—but untested—explanation for these findings is that the antioxidant combination may have reduced the redox-sensitive thiol oxidation of Keap1, thereby attenuating Nrf2 nuclear translocation and downstream cytoprotective gene expression. However, the Vassilakopoulos et al. study did not measure intracellular redox state, Keap1 oxidation, Nrf2 translocation, or Nrf2 transcriptional activity. This interpretation therefore represents a plausible mechanism derived from independent cell-biology literature rather than a direct explanation of the experimental findings.

It is important not to equate attenuation of an acute exercise-induced IL-6 response with impaired physiological adaptation. Exercise-induced IL-6 functions as a myokine with metabolic roles that differ substantially from chronically elevated inflammatory IL-6. The functional consequences of blunting acute cytokine responses require the direct measurement of training adaptation outcomes rather than inference from cytokine concentrations alone. This mechanistic insight suggests that rather than being absolutely contraindicated, chronic high-dose antioxidant supplementation may attenuate selected exercise-induced adaptations under certain conditions, depending on compound, dose, timing, nutritional status, training status, and the outcome measured [8,26].

### 7.3. The Gut–Mucosal Axis: A Hypothesis-Generating Integrative Framework

The changes in salivary sIgA observed after high-intensity exercise are not necessarily an isolated local event; they may reflect systemic shifts in immune trafficking and barrier integrity. The following is presented as an integrative hypothesis connecting individually documented physiological responses, none of which has been demonstrated as a complete pathway in the exercise contexts discussed.

High-intensity exercise can reduce splanchnic blood flow, and this reduction has been directly documented in healthy humans during exercise, with associated gut dysfunction [32,33]. Under conditions where splanchnic hypoperfusion is sufficient, intestinal barrier integrity may be transiently compromised, as demonstrated in human and animal studies of exercise-associated gastrointestinal permeability [34]. If luminal lipopolysaccharide (LPS) translocates into the portal circulation, endotoxin may bind to CD14/TLR4 on hepatic Kupffer cells, a pathway documented in experimental models showing that LPS increases CD14 and TLR4 expression in Kupffer cells in a dose- and time-dependent manner, with associated TNF-α and IL-6 responses [35]. Whether such endotoxemia occurs to a physiologically meaningful degree in the exercise protocols discussed here, and whether it amplifies HPA-axis activation sufficiently to affect MALT B-cells, has not been established. This integrated sequence therefore remains a hypothesis for future investigation rather than a demonstrated mechanism.

The proposal that HIIT-induced ABCA1/reverse cholesterol transport reduces systemic inflammation, enhances HPA-axis resilience, and thereby preserves mucosal immunity is particularly speculative. High-intensity interval training has well-documented cardiometabolic benefits, including the upregulation of reverse cholesterol transport elements. Whether these systemic adaptations modify the neuroendocrine or mucosal immune responses to subsequent exercise bouts is currently unknown and represents a hypothesis for future investigation rather than an established mechanism. The previously proposed concept of a “cortisol threshold” is not operationally defined and has been removed.

### 7.4. Compound-Specific Mechanisms: Astaxanthin’s Kinetics Versus Melatonin’s Conditional Redox Activity

The evidence reviewed in Section 4 underscores that these interventions are therapeutically non-interchangeable. Astaxanthin [19,20] possesses a distinctive molecular architecture: its polar end groups anchor in the phospholipid bilayer, while its polyene backbone spans the membrane’s hydrophobic core. X-ray diffraction studies have shown that astaxanthin preserves membrane structure and exhibits significant antioxidant activity (approximately 40% reduction in lipid hydroperoxide levels) in polyunsaturated fatty acid-enriched membranes, whereas apolar carotenoids such as β-carotene and lycopene disorder the bilayer and exert pro-oxidant effects [36]. These findings indicate that astaxanthin’s membrane interactions differ qualitatively from those of other carotenoids, a property that may contribute to its antioxidant behavior. However, direct comparative evidence in human exercise settings is lacking, and this proposed advantage should be regarded as mechanistically plausible rather than established.

In vitro and animal studies have suggested that astaxanthin modulates the Nrf2 and NF-κB signaling pathways, including their crosstalk [37]. However, the Baralić et al. study did not measure Nrf2 translocation, NF-κB activity, IKKβ signaling, or any other molecular endpoint. The proposal that these mechanisms explain the increased resting sIgA observed in that trial is therefore speculative and remains to be tested directly.

Conversely, melatonin provides a cautionary paradigm in conditional redox behavior [24]. Melatonin is a pleiotropic signaling molecule with context-dependent redox-modulating, endocrine, and immune effects, rather than a conventional direct antioxidant. One proposed—but not directly tested—explanation for these paradoxical findings involves iron-dependent melatonin metabolism. In the presence of redox-active iron, melatonin can be metabolized to AFMK (N1-acetyl-N2-formyl-5-methoxykynuramine), which has been reported to generate pro-oxidant intermediates under certain conditions. However, the Rigamonti et al. trial did not measure labile iron, AFMK, or iron-dependent redox chemistry. Moreover, elevated ferritin should not be interpreted as direct evidence of increased redox-active iron, since ferritin is an acute-phase protein influenced by inflammation and metabolic status. This mechanism therefore remains speculative. The absence of the expected IL-6 reduction in the melatonin group should not be interpreted as proof that melatonin negated the metabolic benefits of the weight-reduction program. Metabolic outcomes were not directly assessed in a manner that permits this conclusion. What can be stated is that melatonin did not produce the same IL-6 trajectory as the placebo in this specific population and context. This context-dependency suggests that the same compound that may improve T-cell proliferation in lean athletes may have different effects in obese individuals with systemic low-grade inflammation.

### 7.5. Neuroimmune Modulation: A Condensed Perspective

Psychological stress, sleep quality, recovery status, and recent illness may modify exercise-related mucosal immune responses. These factors should be considered alongside physical training metrics in monitoring protocols. However, the mechanistic pathways linking neuroimmune modulation to salivary IgA remain to be established, and findings from post-infectious populations should not be generalized to healthy athletes without qualification. Future monitoring protocols may consider integrating perceived stress and cognitive fatigue alongside salivary sIgA and FRAP, but this remains a hypothesis-generating proposal.

### 7.6. Summary of Mechanistic Integration

In summary, the exercise–redox–mucosal interplay has been proposed to be governed by a four-dimensional network: (1) neuroendocrine signaling (cortisol/GR and sympathetic outflow) may contribute to suppressed pIgR transcription and reduced acinar fluid secretion; (2) redox-sensitive transcription factors (Nrf2/NF-κB) could be involved in determining whether the exercise stimulus produces adaptive mitochondrial biogenesis or sustained systemic inflammation, with exogenous antioxidants acting as potential modulators; (3) splanchnic hypoperfusion and LPS translocation during high-intensity efforts may contribute to a gut-derived inflammatory trigger that could amplify HPA activation; and (4) cognitive-emotional status (neuroimmune axis) may moderate the set-point at which these pathways engage. These proposed mechanisms are hypothesis-generating and have not been directly demonstrated in the exercise contexts discussed. The four-dimensional framework is intended to organize future research and monitoring, not to represent a validated diagnostic or prescribing algorithm. Personalized strategies may consider assessing not just plasma redox panels, but also markers of gut permeability, salivary cortisol patterns, and self-reported stress indices to determine an athlete’s individual mucosal-response and recovery profile.

## 8. Limitations of the Current Evidence

- Most intervention trials are small and short, often lasting only several weeks.
- Many studies enroll young, healthy men, limiting generalizability to women, master’s athletes, adolescents, and athletes with metabolic disease.
- Exercise protocols vary widely, making cross-study comparisons difficult.
- Redox biomarkers are heterogeneous and do not represent a single biological construct.
- sIgA methods vary substantially in collection, normalization, timing, and reporting.
- Few trials simultaneously measure exercise dose, redox biomarkers, sIgA, infection outcomes, dietary intake, and recovery variables.
- Many supplement trials use multi-ingredient formulations, preventing attribution to one active compound.
- Associations between low sIgA and respiratory illness should not be interpreted as proof that manipulating sIgA alone prevents infection.
- Genetic and multi-omics personalization remains investigational rather than a validated clinical pathway.
- The proposed four-dimensional framework has not been prospectively validated.

## 9. Future Research Priorities

The next generation of studies should move beyond isolated antioxidant trials toward integrated, longitudinal designs. A useful trial would repeatedly measure training load, dietary intake, sleep and recovery, salivary sIgA secretion rate, selected salivary and blood redox markers, inflammatory mediators, and clinically confirmed respiratory infections. Studies should include both sexes, different age groups, multiple sports, and athletes with distinct training statuses.

Methodological standardization is particularly important. Investigators should prespecify saliva collection time, hydration status, food and fluid restrictions, flow-rate normalization, assay methods, and post-exercise sampling windows. Redox panels should also be selected according to a defined biological question rather than reported as an undifferentiated list of “antioxidant” measures.

Future studies examining gut permeability should use validated, complementary approaches rather than relying on a single surrogate marker. Plasma LPS-binding protein reflects host exposure or response to microbial products rather than directly measuring epithelial permeability. Commercial assays for circulating zonulin have important analytical-specificity concerns. Where feasible, multi-marker approaches incorporating functional permeability tests alongside biochemical markers should be used.

Cortisol measurements in exercise studies should distinguish among acute exercise-induced cortisol responses, diurnal cortisol slope, and the cortisol awakening response. These measurements address different physiological processes and should not be grouped indiscriminately as “cortisol profiling”.

Future trials should compare food-based dietary patterns with isolated supplements, test dose–response relationships, and distinguish adaptation-oriented training from competition and recovery phases. Larger factorial trials could determine whether an antioxidant has greater value in athletes with low baseline antioxidant capacity or during periods of accumulated training stress. Finally, infection outcomes—not merely biomarker changes—should be included whenever the stated goal is the preservation of immune health.

## 10. Conclusions

Exercise exerts hormetic effects on redox biology and mucosal immunity, with moderate doses promoting adaptive signaling and excessive or poorly recovered doses producing oxidative and immune perturbation. Salivary sIgA is a useful, non-invasive marker of mucosal immune status, but its interpretation requires attention to flow rate, secretion rate, and methodological standardization. Current evidence does not support routine high-dose antioxidant supplementation. Instead, a food-first approach, correction of nutrient inadequacy, adequate recovery, and selective supplementation—when the context and evidence justify it—represent the most defensible strategy. Astaxanthin, β-glucans, polyphenol formulations, and sesame show promise but require larger trials linking redox changes to clinically meaningful mucosal outcomes. Melatonin illustrates the importance of metabolic context. Personalized antioxidant strategies should be framed as dynamic risk management rather than precision prescribing. The goal is not to eliminate exercise-induced ROS, but to preserve the redox signaling required for adaptation while minimizing conditions in which cumulative stress exceeds the recovery capacity.

## Figures and Tables

**Figure 1:**
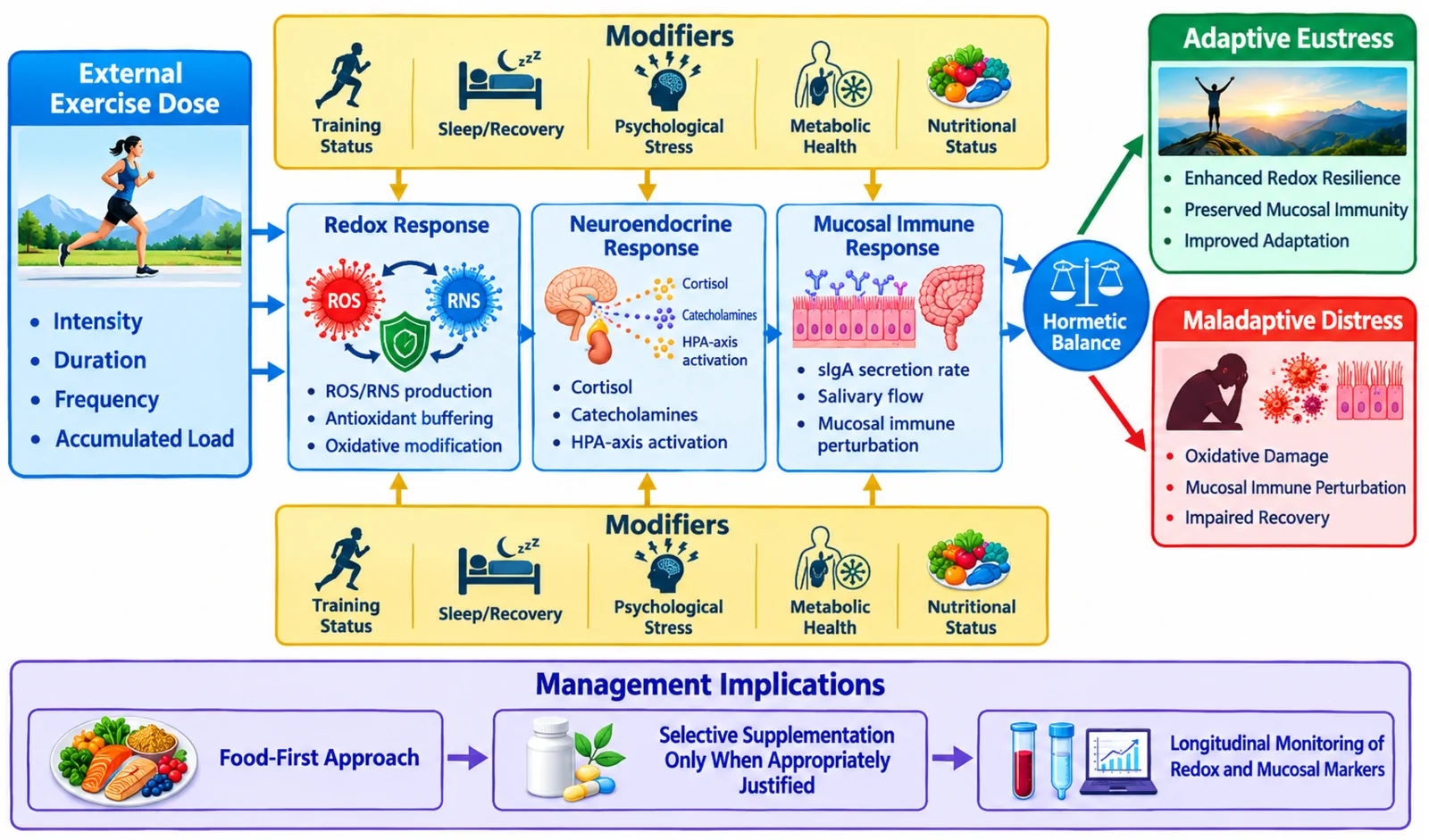
Integrative framework for exercise, redox biology, mucosal immunity, and recovery context.

**Table 1:** Summary of key human studies on exercise, redox status, mucosal immunity, and nutritional interventions.

Study (Year)	Population	Sample Size	Design	Exercise Model	Intervention	Dose/Duration	Redox Outcome	Mucosal/Immune Outcome	Main Limitation
Uchino et al. (2024) [10]	Healthy untrained young men	12	Randomized crossover	Cycling at 35/55/75% VO_2_max × 30 min; 55% × 30/60/90 min	None	N/A	Not assessed	Lower percentage change in sIgA secretion rate at 75% vs. 55% VO_2_max (30 min) and at 90 min vs. 30 min (55% VO_2_max)	Small sample; exclusively male; untrained; between-condition comparisons only
Baralić et al. (2015) [19]	Trained male soccer players	40	RCT	Regular training	Astaxanthin	4 mg/day × 90 days	↓ Prooxidant-antioxidant balance	↑ Resting sIgA; ↓ neutrophils, hs-CRP	Small sample; single study
Asghari et al. (2025) [20]	Professional male water polo players	24	RCT	HIIE: 5 × 50 m sprints	Astaxanthin + cold-water immersion	25 mg/day × 14 days	Not directly assessed	↑ IgA recovery; ↓ IL-6	Combined intervention; not sIgA-specific
König et al. (2026) [21]	Healthy moderately active men	39	RCT, double-blind, placebo-controlled	Two standardized HIIT sessions	β-glucans + micronutrients	8 weeks	↑ FRAP; ↑ SOD at 60 min	↓ TNF-α; ↓ URTI incidence/duration	Multi-component formulation
Huang et al. (2026) [22]	Adolescent athletes (15–17 y)	80	RCT, systems biology	12-week high-intensity training	Polyphenol mix (quercetin, EGCG, resveratrol, curcumin)	1200 mg/day × 12 weeks	Nrf2-mediated responses	Maintained CD4^+^/CD8^+^; sIgA ↓ only 6.5% vs. 29.3% placebo	Adolescent population; multi-compound
Jafari et al. (2026) [23]	Semiprofessional athletes	140 (4 RCTs)	Systematic review	Aerobic exercise	Sesame (oil/extract/capsule/seed)	≥4 weeks	↑ GPx, SOD, Vit A/C/E; ↓ MDA	↓ IL-6, IL-1β	Cytokine changes partly attributable to exercise
Rigamonti et al. (2024) [24]	Adults with obesity	18	RCT	Acute exercise + BWRP	Melatonin	2 mg/day × 14 days	↑ d-ROMs; ↑ HbSSG (paradoxical)	Abolished BWRP-induced ↓ IL-6	Small sample; obesity context
Vassilakopoulos et al. (2003) [17]	Healthy untrained adults	6	Original research	45 min cycling, 70% VO_2_max	Vit E/A/C + allopurinol + NAC	60 days/15 days/3 days	Not directly assessed	Abolished TNF-α/IL-1β; ↓ IL-6	Very small sample; pharmacological doses
Robson et al. (2003) [18]	Endurance-trained adults	12	RCT, crossover	2 h run, 65% VO_2_max	β-carotene + Vit C + Vit E	7 days	↑ Plasma vitamins	↑ Neutrophil oxidative burst	Small sample

Abbreviations: BWRP, body weight reduction program; d-ROMs, derivatives of reactive oxygen metabolites; EGCG, epigallocatechin-3-gallate; FRAP, ferric reducing antioxidant power; GPx, glutathione peroxidase; HbSSG, erythrocytic glutathionyl-hemoglobin; HIIE, high-intensity interval exercise; HIIT, high-intensity interval training; hs-CRP, high-sensitivity C-reactive protein; IL, interleukin; MDA, malondialdehyde; NAC, N-acetylcysteine; RCT, randomized controlled trial; sIgA, secretory immunoglobulin A; SOD, superoxide dismutase; TNF-α, tumor necrosis factor-alpha; URTI, upper respiratory tract infection; Vit, vitamin.

## Data Availability

Data sharing is not applicable to this article as no new data were created or analyzed in this study.
